# Immune Disregulation in Cutaneous Squamous Cell Carcinoma of Patients with Recessive Dystrophic Epidermolysis Bullosa: A Single Pilot Study

**DOI:** 10.3390/life12020213

**Published:** 2022-01-30

**Authors:** Angela Filoni, Gerolamo Cicco, Gerardo Cazzato, Anna Bosco, Lucia Lospalluti, Marco Tucci, Antonietta Cimmino, Caterina Foti, Andrea Marzullo, Domenico Bonamonte

**Affiliations:** 1 Section of Dermatology, Department of Biomedical Science and Human Oncology (DIMO), University of Bari ‘Aldo Moro’, Piazza Giulio Cesare 11, 70121 Bari, Italy; jerrycicco.1@gmail.com (G.C.); annabosco.8.5@gmail.com (A.B.); lucia.lospalluti@policlinico.ba.it (L.L.); caterina.foti@uniba.it (C.F.); domenico.bonamonte@uniba.it (D.B.); 2 Section of Dermatology, Perrino Hospital, S.S.7, 72100 Brindisi, Italy; 3 Section of Pathology, Department of Emergency and Organ Transplantation (DETO), University of Bari ‘Aldo Moro’, Piazza Giulio Cesare 11, 70121 Bari, Italy; micasucci@inwind.it (A.C.); andrea.marzullo@uniba.it (A.M.); 4 Section of Medical Oncology, Department of Biomedical Sciences and Clinical Oncology (DIMO), University of Bari ‘Aldo Moro’, Piazza Giulio Cesare 11, 70121 Bari, Italy; marco.tucci@uniba.it

**Keywords:** epidermolysis bullosa, skin cancer, squamous cell carcinoma, genodermatosis

## Abstract

Background: Cutaneous squamous cell carcinoma (cSCC) is one of the most devastating complications of recessive dystrophic epidermolysis bullosa (RDEB). We recently demonstrated a reduction in immune cell peritumoral infiltration in RDEB patients with cSCC, together with a reduction in CD3+, CD4+, CD68+ and CD20 lymphocytes as compared to primary and secondary cSCC in patients without RDEB. Recently, new molecules, such as high mobility group box 1 (HMGB1), T cell immunoglobulin, mucin domain 3 (TIM-3) and Heme oxygenase-1 (HO-1), have been shown to play a role in antitumoral immunity. Objective: Patients with RDEB are known to be at increased risk of developing skin cancers, including the dreaded squamous cell carcinoma of the. Tendentially, cSCCs that arise in the context of EBDR are more aggressive and lead to statistically significant bad outcomes compared to cSCCs developed on the skin of patients without EBDR. In an attempt to study the microenvironment of these lesions, we conducted an immunohistochemical analysis study of proteins that could be actively involved in the genesis of this type of malignant neoplasms. Methods: In this retrospective study, the OH1-HMGB1-TIM3 activation axis, as correlated to the T lymphocytes cell count, was assessed in biopsy samples from 31 consecutive cases consisting of 12 RDEB patients with cSCC, 12 patients with primary cSCC and 7 RDEB patients with pseudoepitheliomatous cutaneous hyperplasia. Parametric Student’s *t*-test was applied for normally distributed values, such as CD4+ and CD8+, and non-parametric Mann–Whitney test for non-normally distributed values, such as HMGB-1, TIM-3 and HO-1. Results: In RDEB patients with cSCC and with pseudoepitheliomatous hyperplasia, the expression of CD4 T helper lymphocytes was lower than in the peritumoral infiltrate found in primary cSCC. CD8 cytotoxic T lymphocytes were increased in primary cSCC compared to the other two groups. An increased HMGB1 expression was evident in both primary and RDEB cSCC. TIM3 expression was higher in RDEB patients with cSCC compared to the other two groups. A significantly reduced immunohistochemical expression of HO-1 was evident in the tumoral microenvironment of cSCC-RDEB as compared to primary cSCC. Conclusions: These data suggest that a reduced immune cell peritumoral infiltration in RDEB patients could be responsible, in the complexity of the mechanisms of carcinogenesis and host response, of the particular aggressiveness of the cSCC of RDEB patients, creating a substrate for greater local immunosuppression, which, potentially, can “open the doors” to development and eventual metastasis by this malignant neoplasm.

## 1. Introduction

Recessive dystrophic epidermolysis bullosa (RDEB) is an invalidating genodermatosis characterized by skin and mucosa fragility and blister-formation. One of the most devastating complications of this disease with high morbidity and mortality rates is cutaneous squamous cell carcinoma (cSCC). It has been estimated that patients with RDEB have a 70-fold higher risk of developing cSCC compared to unaffected individuals, the mean age at diagnosis being 36 years versus 80 years in non-EB patients [1]. Multiple causes underlie this greatly increased susceptibility, largely attributable to the genetic condition itself. In fact, scientific evidence points to the role of recurrent bacterial infections [2], lack of collagen VII [3], as well as general [4] and local [5] impairment of the immune defenses, creating a favorable microenvironment for the growth of this tumor. In recent work, we demonstrated a reduced immune cell peritumoral infiltration in patients with RDEB, with a significant reduction in CD3+, CD4+ and CD68+ in RDEB patients with cSCC compared to primary cSCC in patients without RDEB, as well as a significant reduction in CD3+, CD4+, CD8+ and CD20+ in RDEB patients with cSCC compared to non-RDEB patients with secondary cSCC (post-burns and post-radiotherapy) [5]. Recently, new molecules, such as high mobility group box 1 (HMGB1), T cell immunoglobulin, mucin domain 3 (TIM-3) and Heme oxygenase-1 (HO-1), have been shown to play a role in antitumoral immunity.

HMGB1 is a non-histone nuclear protein, the prototype of the so-called damage-associated molecular pattern or alarmins [6], whose levels have been demonstrated as significantly elevated in patients affected by RDEB, in both blisters fluid and serum [7], and positively correlated to the Birmingham Epidermolysis Bullosa Severity Score [8]. On the other hand, in cSCC non-RDEB, HMGB1 overexpression in immunohistochemistry seems to be correlated to tumor progression, owing to the close link between this mediator and the tumor’s invasive and metastatic potential [9,10].

TIM-3, a transmembrane glycoprotein member of the TIM genes family, is numbered among the so-called immunological checkpoints, or co-inhibitory receptors whose prototypes are CTLA-4 and PD-1 [11], and is expressed on Th1, Th 17, CD8+ cytotoxic lymphocytes, on NK cells, and on cells of a myeloid lineage [12,13]. By binding to HMGB1 and all its ligands, it has an inhibitory action on T lymphocytes, suppressing Th1 and Th17 responses [13,14,15] as well as reducing the CD8+ lymphocytes proliferation and cytokines production [12]. TIM-3 would thus play a specific inhibitory role, especially in the context of antitumoral immunity. Indeed, elevated levels of TIM-3 on T CD8+ lymphocytes have been associated with tumor progression and a worse prognosis in tumors, such as cSCC and malignant melanoma [15,16].

HO-1 is an inducible microsomal enzyme; scientific evidence has highlighted an inverse relation between HO-1 and HMGB1 levels. Since the progression of cSCC is correlated with HMGB1 [9] overexpression, it is plausible to suppose that high levels of HO-1 can contribute to stem neoplasia growth and metastasis.

The aim of our study was, therefore, to assess the OH1-HMGB1-TIM3 activation axis in biopsy samples from cSCC patients affected by RDEB, from primary cSCC in non-RDEB subjects and from pseudoepitheliomatous cutaneous hyperplasia in RDEB patients.

## 2. Materials and Methods

A retrospective study was made of 31 consecutive cases: 12 cases of cSCC in patients affected by severe RDEB (Group 1) were compared with 12 consecutive cases of primary cSCC in non-RDEB patients (Group 2) and 7 cases of RDEB patients affected by pseudoepitheliomatous cutaneous hyperplasia (Group 3). The cSCC site classification was based on that of the National Comprehensive Cancer Network (NCCN) of 2017 [17].

Excisional biopsy samples (obtained between 2018 and 2021) were subjected to histological assessment after fixation in neutral 10% buffered formalin, dehydration and paraffin-embedding. From the paraffinized blocks, 5 µm sections were taken, deparaffinated, rehydrated and routinely stained with hematoxylin-eosin. Immunohistochemistry was performed using antibodies against the following markers: anti-CD4: mouse monoclonal Ab (mAb), code M7310, (DAKO, Carpinteria, CA, USA), dilution 1:50; anti-CD8: mouse monoclonal Ab (mAb), code NCLCD8-295, (Novacastra Laboratories Ltd., Newcastle), dilution 1:50; anti-HMGB1: rabbit polyclonal Ab, Ab18256 (Abcam, Cambdridge, United Kingdom), dilution 1:1000; anti-TIM-3: rabbit polyclonal Ab, GTX 54117 GeneTex, dilution 1:100; Anti-Heme Oxygenase-1 (HO-1): rabbit polyclonal Ab, GTX101147 GeneTex, dilution 1:500.

The blocks had been submitted to antigen unmasking using the PT-LINK (DAKO) device, with EDTA (EnVision Flex, target retrieval solution, High Ph (50×), DAKO) for antibodies against CD4, CD8 and Citrate (EnVision Flex, target retrieval solution, Low Ph (50×), DAKO) for the HMGB1, TIM3 and OH1 antibodies.

The immunohistochemical reactions were evaluated, investigating the CD4 and CD8 markers cell density by counting positive cells in 10 fields (HPF) for each clinical case. Each field, 140 microns long by 110 microns wide, with a total amplitude of 15,400 microns squared, was examined at 400× magnification.

The expression of HMGB1 [18], TIM-3 [19] and HO-1 [20,21] was assessed by highlighting the chromogen signal on the cytoplasm, in the nucleus or extracellular medium of the samples analyzed. For HMGB1, TIM-3 and HO-1, a score was assigned, summing the different degrees of staining intensity (grade 0 = no staining; grade 1 = weak staining; grade 2 = moderate staining; grade 3 = intense staining) plus the score for the percentage extension of the mass (score 0: <1%; score 1: 1–25%; score 2: 26–50%; score 3: 51–74%; score 4: ≥75%). The final score (sum of the 2 previous scores) was considered high if >3 versus low if ≤3. The peritumoral cellular infiltrate was assessed in the same way. The preparations were examined by two dermatopathologists with high confidence with skin lesions (A.C. and G.C.), and if they disagreed, a third dermatopathologist (C.A.) was discussed and broadened the vision. The study was reviewed and approved by the local ethical committee.

### Statistical Analysis

The mean and standard deviation values for the 10 fields were recorded for each patient. Normal distribution was assessed with the Kolmogorov–Smirnov test. Parametric Student’s *t*-test was applied for normally distributed values, and non-parametric Mann–Whitney test for non-normally distributed values. TIM3, HMGB1 and OH-1 values resulted non-parametric, while CD4 and CD8 values were parametric. A comparison was made of the means in the single groups and then between the three study groups. A value of *p* ≤ 0.05 was set as statistically significant. All statistical analyses were made using the Prism 9.0.3 program, GraphPad software, 9.0.1 version, 2021, La Jolla (CA, USA).

## 3. Results

Patients gender, age, lesion sites, size, histological subtype, differentiation grade, thickness, lymphatic and/or vascular invasion and perineural involvement are reported in Table 1.

CD4 expression values were 7.92 ± 1.77 (cells/mm^2^) in Group 1, 54.27 ± 10.57 (cells/mm^2^) in Group 2, and 11.79 ± 3.38 (cells/mm^2^) in Group 3. The difference was statistically significant between Group 1 and Group 2 (*p* = 0.0008) and between Group 2 and Group 3 (*p* = 0.0007), but not significant between Group 1 and Group 3 (*p* = 0.36).

CD8 expression values were 14.27 ± 4.92 (cells/mm^2^) in Group 1, 52.76 ± 10.20 (cell/mm^2^) in Group 2, and 29.13 ± 6.4 (cells/mm^2^) in Group 3, resulting statistically different between Group 1 and Group 2 (*p* = 0.0049), but not between Group 2 and Group 3 (*p* = 0.06), nor between Group 1 and Group 3 (*p* = 0.092).

**Figure 1:**
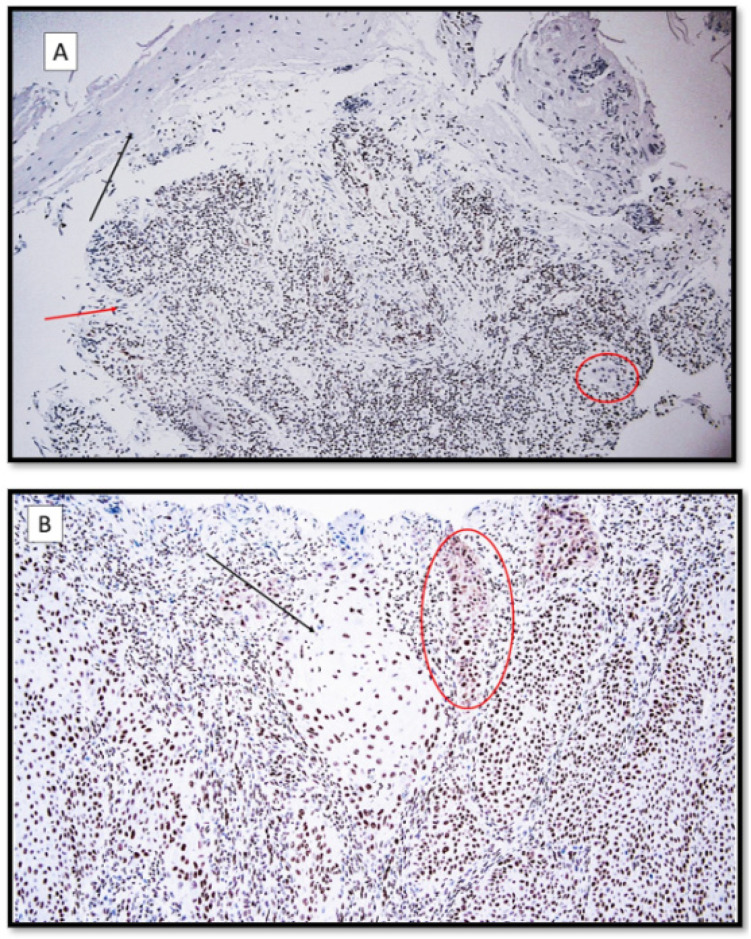
(**A**) HMGB-1 immunoexpression, localized predominantly in the interstitial space and inflammatory peritumoral component in a patient with recessive dystrophic epidermolysis bullosa cSCC. Note the faint nuclear and cytoplasmic staining of the neoplastic masses (HMGB-1 antibodies, original magnification 100×). Black arrow: ulcerated epidermis; red arrow: moderately differentiated cutaneous squamous cell carcinoma (cSCC). In the red circle, there is a neoplastic token. (**B**) HMGB-1, predominantly nuclear immunoexpression with a focally cytoplasmic localization in a patient with primary cSCC without EBDR. Black arrow: large masses of well-differentiated nuclear-positive cSCC for HMGB1. Red circle: neoplastic tokens entirely positive for immunostaining. (HMGB-1 antibodies, original magnification 200×).

**Figure 2:**
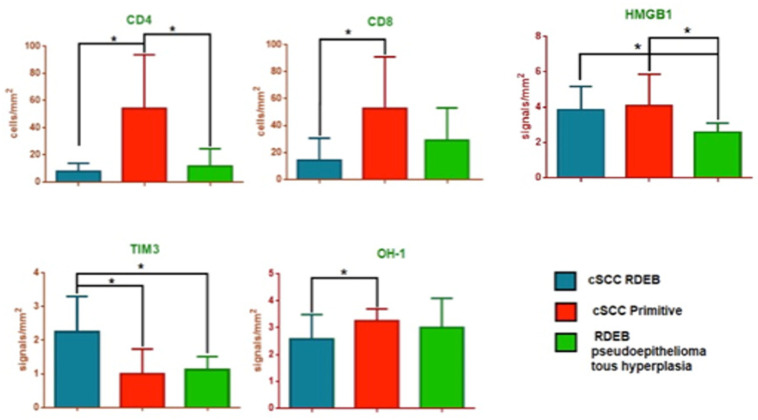
CD4, CD8, high mobility group box 1 (HMGB1), T cell immunoglobulin and mucin domain 3 (TIM-3) and Heme oxygenase-1 (HO-1) analysis in RDEB patients with cutaneous squamous cell carcinoma (cSCC) (Group 1) compared to non-RDEB patients with primary cSCC (Group 2) and to RDEB patients with pseudoepitheliomatous cutaneous hyperplasia (Groups 3). * stastically significant.

HMGB1 expression values were 3.83 ± 0.38 (signal/mm^2^) in Group 1, 4.08 ± 0.51 (signal/mm^2^) in Group 2, and 2.57 ± 0.20 (signal/mm^2^) in Group 3, eliciting significant differences between Group 1 and Group 3 (*p* = 0.03), and between Group 2 and Group 3 (*p* = 0.044) (Figure 1 and Figure 2).

**Figure 3:**
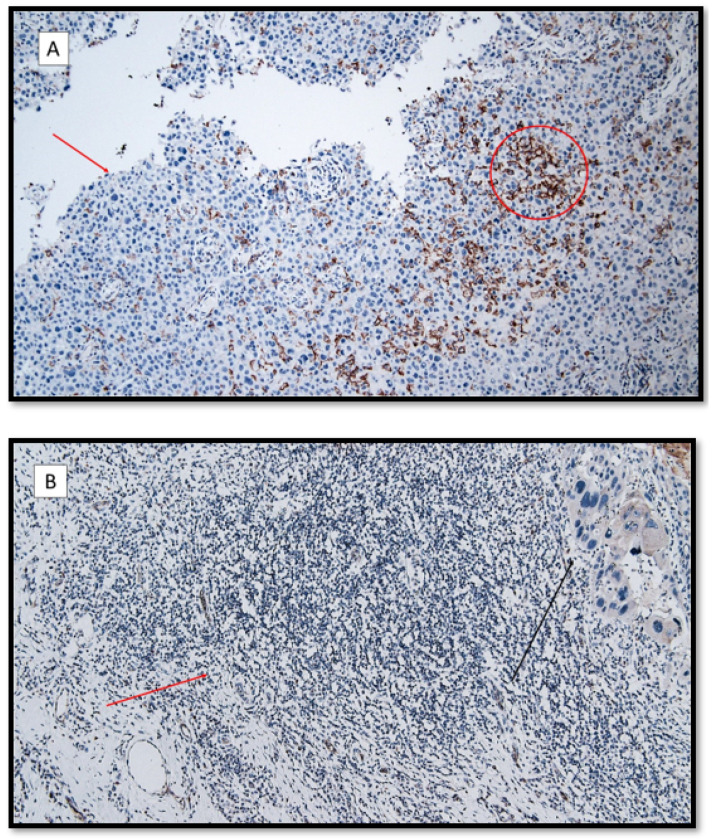
(**A**) TIM-3 immunoexpression in the lymphocytic T component (red circle) and keratinocytes (red arrow) in a patient with recessive dystrophic epidermolysis bullosa cSCC. (TIM-3 antibodies, original magnification 100×). (**B**) Almost totally negative TIM-3 immunoexpression in the inflammatory component in a patient with primary cSCC (red arrow). The black arrow shows an infiltrative component of a poorly differentiated cutaneous squamous cell carcinoma without EBDR. (TIM-3 antibodies, original magnification 100×).

TIM3 expression values were 2.25 ± 0.30 (signal/mm^2^) in Group 1, 1.00 ± 0.21 (signal/mm^2^) in Group 2, and 1.14 ± 0.14 (signal/mm^2^) in Group 3, eliciting significant differences between Group 1 and Group 2 (*p* = 0.0028), and between Group 1 and Group 3 (*p*= 0.016) (Figure 2 and Figure 3).

**Figure 4:**
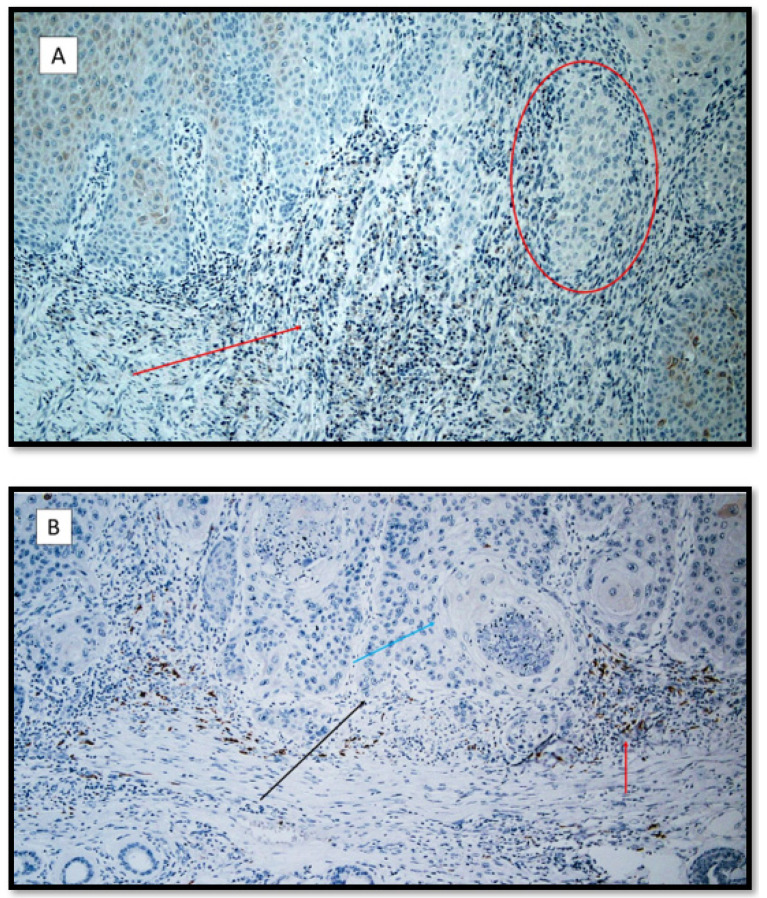
(**A**) Heme-oxygenase 1 immunostaining: note the rare inflammatory elements and positive keratinocytes in a patient with recessive dystrophic epidermolysis bullosa cSCC (red arrow). The red circle shows a neoplasm token almost entirely negative for HO-1. (Antibody for HO-1, original magnification 100×). (**B**) Heme-oxygenase 1 immunostaining: note the higher expression in the cSCC inflammatory microenvironment. (red arrow). The black arrow shows the neoplastic component of this moderately differentiated cSCC; the blue arrow points to a horny pearl. (Heme-oxygenase 1 immunostaining, original magnification 100×).

Heme Oxygenase-1 expression values were 2.58 ± 0.26 (signal/mm^2^) in Group 1, 3.25 ± 0.13 (signal/mm^2^) in Group 2 and 3.00 ± 0.44 (signal/mm^2^) in Group 3, eliciting significant differences between Group 1 and Group 2 (*p* = 0.031) (Figure 2 and Figure 4).

## 4. Discussion

Epidermolysis bullosa comprises a group of genetically determined disorders characterized by blistering of the skin and mucosae [1,2,3,4]. The dystrophic form of EB is characterized by scarring, nail changes and milia and has either autosomal recessive or dominant inheritance [4]. The recessive form, so-called RDEB, is characterized by the presence of bullae that, usually, are present at birth or appear in early infancy [3,4], later-onset being exceptional. Other pathological manifestations are determined at the level of the cornea (corneal erosions) and nail (nail dystrophy), but the most fearful sequela is represented by squamous skin carcinoma [4,5,6,7]. These forms of cSCC sometimes are multiple and may appear well differentiated histologically, but, in keeping with other scar carcinomas, clinically, these tumors behave aggressively, recur locally and often metastasize [8,9,10,11,12,13]. Although scientific research has progressed at a rapid pace, the rarity of EBDR and the difficulty in finding large cohorts of patients have made it difficult to study and investigate the mechanisms of pathogenesis.

While studying the tumor microenvironment of this patient population, we found that in RDEB patients affected by cSCC, the expression of CD4 lymphocytes is lower than in the peritumoral infiltrate found in primary cSCC and in RDEB patients with pseudoepitheliomatous hyperplasia. This finding is coherent with our previous study, describing a reduced number of CD3 and CD4 T helper cells in cSCC in RDEB patients as compared to patients with primary or secondary cSCC and cSCC in renal transplant patients [5]. Moreover, the CD4 peritumoral component in primary cSCC showed a high expression of T helper lymphocytes, in agreement with the literature [22,23].

In the present study, CD8 cytotoxic T lymphocytes were increased in primary cSCC compared to the other two groups, but significantly different only when comparing RDEB patients affected by cSCC with non-RDEB patients affected by primary cSCC. These results could be correlated to the greater expression of TIM3 in the peritumoral microenvironment in RDEB patients cSCC. The peritumoral expression of cytotoxic CD8 was very strong in primary cSCC, in agreement with the literature [22,23]. In our study, there was no statistically significant difference between cSCC in RDEB patients and patients with PH without clear cSCC. To explain this result, a technical problem must be considered. In fact, the skin of patients affected by EB is profoundly altered, both from a functional and morphological point of view; therefore, biopsies are not always easy to perform, and it must be considered that the histological differential diagnosis between IP and cSCC on RDEB is never simple. Therefore, it is plausible that the disease (RDEB) reduces the presence of CD4 and CD8 T lymphocytes, but with differences that are not significant.

HMGB1 expression was not different between the cSCC-RDEB group compared to the primary cSCC group; indeed, both groups showed a very strong HMGB1 expression in the peritumoral microenvironment. An increased expression of HMBG1, a marker of cellular necrosis [23], is observed in a wide variety of tumors, especially skin cancers [9,24]. In fact, HMGB1 has a mitogenic action on many cell types [25], exerts pro-angiogenetic activities [26,27,28] and determines up-regulation of the intercellular adhesion molecule (ICAM)-1 and vascular adhesion molecule (VCAM)-1 [29]. HMGB1 also has effects on both innate immunity cells (dendritic cells (DCs), monocytes/macrophages), and lymphoid cells. By binding to the Toll-Like Receptor 4 and TIM3, it suppresses the proliferation of T lymphocytes and the production of IFNγ, indicating that the co-stimulatory signal of T Cell Receptor is abrogated by HMGB1 [30].

Our data are in agreement with the literature describing an increased HMGB1 expression in both primary cSCC [31,32] and RDEB patients cSCC [33]. In a murine skin model of RDEB, HMGB1 expression was higher in the cSCC than in the non-neoplastic RDEB skin; the latter, in turn, was higher than in control non-RDEB skin. Moreover, HMGB1 levels are reported to be correlated to the Birmingham Epidermolysis Bullosa Severity score [34]; this cytokine seems to be a promoter of skin carcinogenesis [31] and to play a role in hindering the healing of skin wounds [35].

HMGB1 binds to TIM-3 expressed on Dendritic Cells, inhibiting their activation [12], but it has been postulated that the HMGB1/TIM-3 complex may also directly downregulate T cell responses by binding CD8+ Treg that, in turn, suppress the proliferation of effector T cells [13,36]. In this way, HMGB1 could trigger inhibitory TIM-3-dependent pathways in both innate immunity cells and T cells [15]. In fact, TIM-3 is widely expressed on T antigenic tumor-specific lymphocytes, in peripheral blood and in tumor-infiltrating lymphocytes (TIL), both CD4+ and CD8+ [37], and overexpressed on so-called exhausted T cells (especially CD8+ and CD4+ Treg Foxp3+) in tumor settings [12,15]. T lymphocytes exhaustion is directly implicated in an immunosuppressive state in cancer patients [13].

In our study, TIM3 expression was higher in RDEB patients affected by cSCC compared to the other two groups; this finding has never previously been described in EB patients. This result could also be correlated with the more aggressive nature of cSCC in RDEB patients than in patients with primary cSCC forms. Thus, the higher TIM3 immunoexpression found in RDEB patients with cSCC than in patients with primary cSCC could likely be due to a downregulation of T-mediated responses by TIM3 [24,38]. In view of the extracellular expression of HMGB1 and overexpression of TIM3 on the immune cell membrane, it may be postulated that the HMGB1-TIM3 complex has a role in generating an immune deficit in the tumor surveillance mechanism, allowing tumoral cells to escape the antitumoral response.

Our results also show a significantly reduced immunohistochemical expression of Heme oxygenase-1 (OH-1) in the tumoral microenvironment of cSCC-RDEB compared to primary cSCC. In fact, data in literature seem to suggest an inverse correlation between HO-1 and HMGB1. Studies of immortalized human keratinocytes have demonstrated that an increased HO-1 activity, stimulated by the administration of hemin, a known HO-1 inducer, is able to inhibit the release of UV-correlated HMGB1 in these cells [39]. Therefore, it may be hypothesized that low levels of HO-1 may create a favorable microenvironment for tumoral growth and progression [39].

The possible protective role of HO-1 against carcinogenesis is supported in an experimental study in an HO-1 knock-out mouse model, in which a progressive increase in the size and aggressiveness of cSCC was described [40].

Our preliminary data confirm a reduction in immune cell peritumoral infiltration in RDEB patients with cSCC, suggesting that immune dysfunction may contribute to the greater cSCC aggressiveness in patients with RDEB.

## 5. Conclusions

Recessive dystrophic epidermolysis bullosa represents a pathology so rare that it represents difficulties in being able to enroll large patient series, and for this reason, works that investigate its etiopathogenetic mechanisms are particularly important. Our paper, albeit with limitations due to the number of the sample and the immunohistochemical investigation conducted alone, represents an effort to understand the biological basis of the development of particularly aggressive neoplasms in this cohort of patients. Further studies with larger case series are needed to shed light on still unclear aspects of this rare but devastating condition.

## Figures and Tables

**Table 1:** Clinical and histopathological characteristics of the cutaneous lesions analyzed.

	RDEB-cSCC (12) Group 1	Primary cSCC (12) Group 2	RDEB Pseudoepitheliomatosus Hyperplasia (7) Group 3	*p* Value
**Gender (Male/Female)**	2/10	8/4	1/6	Group 1 vs. Group 2 0.036 Group 2 vs. Group 3 0.057
**Age (years)**	38.17 ± 12.49	80.58 ± 9.29	31.57 ± 13.43	Group 1 vs. Group 2 <0.0001 Group 2 vs. Group 3 <0.0001
**Location**				NS
**H**	2	3	3	
**L**	10	9	4	
**M**	-	-	-	
**Size**	1.3 cm	2.8 cm	1.2 cm	NS
**Histological subtypes**	12/12 infiltrative pattern	4/12 infiltrative pattern 6/12 expansive pattern 2/12 Exophytic pattern	-	NS
**Differentiation grade**	11/12 poorly differentiated 1/12 moderately differentiated	8/12 moderately differentiated 3/12 poorly differentiated 1/12 well differentiated	-	Group 1 vs. Group 2 0.0007
**Depth (thickness)**	1.65 mm	2.6 mm	-	NS
**Lymphatic/vascular invasion**	6/12	7/12	-	NS
**Perineural involvement**	5/12	5/12	-	NS

Legend. cSCC: cutaneous squamous cell carcinoma, RDEB: recessive dystrophic epidermolysis bullosa. Location based on National Comprehensive Cancer Network classification of 2017: H = mask areas, genitalia, hand and feet; L = trunks and extremities (excluding pretibial, hands, feet, nail units and ankles); M = cheeks, forehead, scalp, neck and pretibial; NS = not significant.

## Data Availability

Not applicable.
